# Studying biological membranes with extended range high-speed atomic force microscopy

**DOI:** 10.1038/srep11987

**Published:** 2015-07-14

**Authors:** Adrian P. Nievergelt, Blake W. Erickson, Nahid Hosseini, Jonathan D. Adams, Georg E. Fantner

**Affiliations:** 1 Laboratory for Bio- and Nano-Instrumentation École Polytechnique Fédérale de Lausanne Batiment BM 3109 Station 17, 1015 Lausanne, Switzerland

## Abstract

High—speed atomic force microscopy has proven to be a valuable tool for the study of biomolecular systems at the nanoscale. Expanding its application to larger biological specimens such as membranes or cells has, however, proven difficult, often requiring fundamental changes in the AFM instrument. Here we show a way to utilize conventional AFM instrumentation with minor alterations to perform high-speed AFM imaging with a large scan range. Using a two—actuator design with adapted control systems, a 130 × 130 × 5 *μ*m scanner with nearly 100 kHz open—loop small-signal Z—bandwidth is implemented. This allows for high-speed imaging of biologically relevant samples as well as high-speed measurements of nanomechanical surface properties. We demonstrate the system performance by real-time imaging of the effect of charged polymer nanoparticles on the integrity of lipid membranes at high imaging speeds and peak force tapping measurements at 32 kHz peak force rate.

In recent years, high-speed AFM (HS-AFM) has been demonstrated to be very powerful for studying biological systems at the nanoscale^1^,^2^. These studies were made possible by a continuous improvement in HS-AFM instrumentation toward even higher scan speeds and better feedback performance^3^,^4^,^5^. Current HS-AFMs are highly specialized, complex instruments tailored to measuring the dynamics of nanoscale systems such as molecular motors^6^, membrane proteins^7^,^8^, or antibodies^9^ with temporal imaging rates of less than one second per image. These measurements have stirred high hopes for advancements in other bio- and nanotechnology fields as well. However, many applications of AFM in cellular biology^10^,^11^,^12^ and materials science^13^,^14^ require scan sizes of 10 *μ*m to 100 *μ*m rather than the hundreds of nanometres of current HS-AFMs. In order to make HS-AFM available to the broad nanotechnology community it is therefore essential to enable large scan ranges in all directions at high speeds^15^,^16^,^17^. In addition, AFM has evolved away from a tool for dedicated experts into a routine tool where many users only operate the instruments occasionally. The high degree of complexity of current HS-AFMs is therefore still prohibitive for broader adoption. A HS-AFM for the general nanotechnology community should not be more difficult to use than a conventional AFM. These two requirements put especially high demand onto the scanner and its control. Increasing the scan size at a fixed scan rate results in an increased tip surface speed. Therefore, the spatial frequency of sample topography translates to higher temporal frequencies when scanning a larger area. Imaging at a larger scan size thus not only puts more burden on the X-Y axes, but also requires a very high bandwidth of the feedback in general and the Z-scanner in particular. In this paper, we describe an approach combining modification of a conventional AFM scanner with model-based control in all three directions. This leads to a total scan range of 130 × 130 × 5 *μ*m in X, Y, and Z respectively, while maintaining a flat small—signal frequency response up to 90 kHz in Z—direction.

Traditionally, the main limitation for scanners has been the excitation of mechanical resonances by the scanner drive signal (see Fig. 1). Piezoelectric tube scanners which are predominantly used in AFM exhibit a large number of different resonant modes, of which the first lateral and the first length extensional modes are of primarily influence on imaging performance (see Fig. 1b).

Lateral resonances are excited when the scan direction is reversed at the end of a line; the sharp turnaround makes the tube structure ring (oscillate) in the direction of the fast scan axis. This ringing is visible as horizontal waves in the image, caused by a coupling of the X-Y resonance into Z-motion due to sample tilt and the well-known scanner bow effect of tube scanners (Fig. 1b inset). Less obvious, but just as severe is that the lateral ringing will severely displace the scan position, making vertical strips in the image appear alternately stretched and compressed^18^ (see original edge displacement in Fig. 1a). The length extensional resonance (Z-ringing) is visible as horizontal ripples that follow sharp steps in the topography.

Control systems have been used extensively in the past to extend the speed of available scanners^19^,^20^. This approach has shown to be a straightforward way to improve the performance of existing mechanical designs with electronics, but is ultimately still limited by the decreasing transducer efficiency at higher frequencies past their resonance. To push the resonance frequencies to higher values, a number of mechanical designs for high—speed large—range scanners have been proposed. Most high—speed scanners are flexure based designs^16^,^21^,^22^ and often use control techniques to deal with resonances^23^. These scanners are fast, but are typically limited in range, as they trade scan range for speed by decreasing the size of the actuators. More recently, feedback using two different Z-actuators has been shown to offer a promising increase in speed^24^,^25^. The tip and the sample are moved with different actuators, where one actuator is lower range but faster. Corresponding control systems have been proposed^26^. This approach is fast, but no longer provides a monolithic scanner and requires the user to interface with two actuators.

We propose a solution for a large range, high—speed system through simple modifications of a commercial system to move the sample with two different actuators in mechanical series and extending the control loop with model-based control for this scanner.

## Mechanical modifications

HS-AFM is primarily enabled on the detection side by the use of small cantilevers with high resonance frequencies^27^ and on the actuation side by scanners with high bandwidth^1^,^3^ together with appropriate controllers^28^. These technologies generally require a complete redesign of the instrument. Recently, we reported an open-source add-on to a commercial instrument (Bruker Multimode 8) that allows the use of very small cantilevers. The replacement AFM head has a detection bandwidth for resonance frequencies up to 20 MHz^29^,^30^ and thereby allows fast topography detection on a standard AFM system.

Here we extend this system by enabling high-speed actuation through a simple addition to the standard piezoelectric tube scanner. We place a secondary stack piezo actuator on top of the tube scanner (see Fig. 2b). The piezo stack has a significantly higher resonance frequency than the tube, due to its small size and higher rigidity. The tube scanner has a scan range of 130 × 130 *μ*m and a Z range of 5 *μ*m. The stack is mounted in a custom flexure housing that acts as a flexure guide for uniaxial Z-motion, fluid sealing of the stack piezo and integrated preload, providing a sample movement of 1.5 *μ*m. A silicone O-ring (Bruker FCO-10) can be seated on the housing for fluid imaging in a closed fluid cell. The sample is mounted on the radial flexure in the top part (titanium grade 5) of the housing (see Fig. 2c).

Using a second actuator on top of the original tube influences the dynamic behaviour of the whole scanner. Instead of the simple harmonic oscillator (SHO) behaviour of a single tube, the system acts as a coupled two mass oscillator (see Fig. 2d) with two inputs and therefore two transfer functions. By frequency domain system modelling (see appendix), we find the tube actuator to behave like an SHO with added mass, while the stiff stack actuator on top can resonantly couple into the much softer tube (Fig. 2e). The lateral resonances of the scanner are only affected insofar as far as that there is additional mass, which slightly lowers the lateral resonance frequency.

## Control systems

Suppression of SHO-like scanner resonances, both lateral as well as axial, has been studied extensively in the past. Using a first order notch filter, the resonant behaviour of the tube can be anticipated and counteracted before the signal is applied to the actuators. Lateral resonances can be nearly completely supressed, and this approach has been shown to offer significant tracking-speed improvements over uncompensated systems, as the scanner can be used to a bandwidth close to the mechanical resonance frequency^31^,^32^,^33^.

Since the combined scanner has two actuators in Z-direction, it can be considered as a multiple—input (two piezo drives) single—output (sample height) system, and can achieve better overall performance by using both inputs. By balancing control authority over two actuators we can achieve the combination of high bandwidth and large range simultaneously. Several schemes exist in literature to utilize two actuators to control for one distance^34^,^35^, of which several have been used for AFM in the past^36^,^37^. The most direct approach to dual actuation with reduced range on one fast actuator is nested PID feedback. A high—frequency primary PID to the fast actuator tracks topography while a secondary slower loop keeps the mean of the fast signal centred. While simple in its implementation, this approach is not robust and cannot deal with coupled dynamics and requires extensive user interaction^38^. Other implementations that use a model based approach for the whole Z-feedback loop can achieve excellent performance and guaranteed stability^39^, but do not leave room to tune the feedback manually to get the best performance for each sample. We therefore chose an approach that is transparent for the AFM controller and does not require any change from standard AFM operating procedures.

To split a signal conservatively across the frequency spectrum, audio systems have long since utilized frequency crossovers. We use the same design to split our signals into low— and high—frequency components. We use the high resonance frequency of the stack actuator to transduce high frequency components, which generally have smaller amplitudes. Low frequency movements are sent to the tube actuator with its longer range.

Models are a requirement for dynamic filtering of dynamics. We use the fact that the AFM itself is a fast, precise sensor that is already in place to measure the dynamics of our system without additional hardware modifications. We can record the transfer functions of both Z actuators by exciting the respective actuator and measuring the deflection of a cantilever in contact mode. Burns *et al.* have presented a method that enables to also measure lateral dynamics directly with the cantilever. By exciting the turnaround and measuring the ringdown in the X—Y to Z coupling, the SHO resonance and damping can be extracted without the use of additional sensors^31^.

The tube roll—off of the cross—over is set to start well below the resonance of the tube (400 Hz) and residual effects of the first order resonance of the tube can be filtered with a simple notch filter at about 8 kHz. The first resonance of the stack actuator can be cancelled in a similar way with a notch filter at ca. 80 kHz. However, the coupling dynamics of the stack actuator into the resonances of the tube is more involved. Using model inversion, we filter the stack signal in elementary blocks, each compensating a resonant coupling (coupling filters) in addition to the notch filter to suppress the stack resonance (Fig. 3a) (See supplementary information for the models used for fitting). All filters are implemented as a sequence of second order sections on field programmable gate arrays (FPGAs) (National Instruments, X-Filter: NI PXI-7851R, Z-Filters: NI 7954R/NI 5781). The measured small—signal transfer functions of the systems as well as their corresponding filters are shown in Fig. 3b. Due to the frequency crossover, the second tube resonance is already strongly attenuated, and does not require an additional notch filter. The resulting small—signal open—loop response of the whole system is flat within ±3 dB to 90 kHz and results in a nearly 20 times faster closed loop response than the unmodified tube scanner could achieve. With our current implementation the system delay, in large part caused by analog—to—digital conversions and digital—to—analog conversions, restricts the non-peaking closed loop operation (Bessel type) to about 35 kHz. For imaging purposes a faster operation can be achieved by allowing some frequency domain peaking.

**Figure 1:**
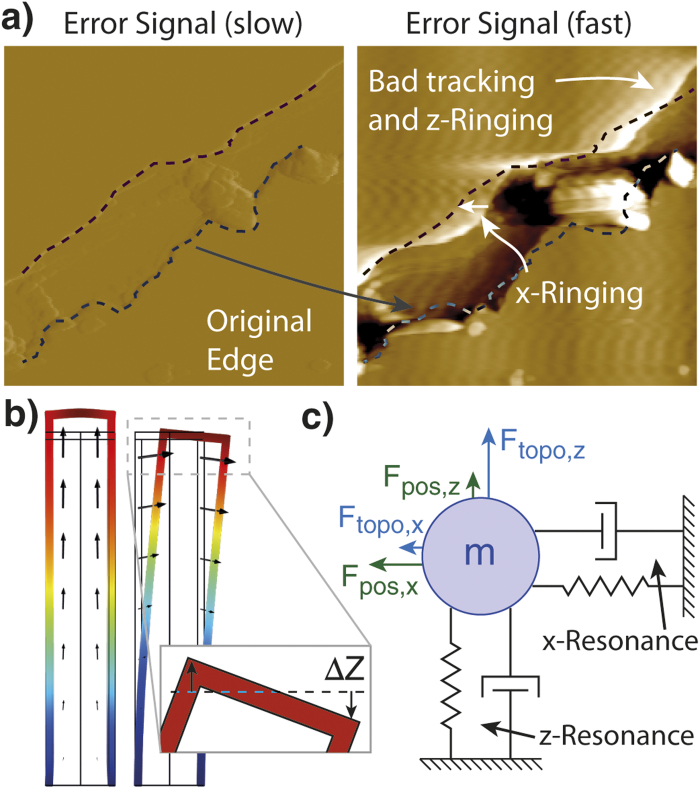
Distortions of AFM image due to scanner resonances at slow scan rates (left image, 4 Hz line rate) and high scan rate (right image, 166 Hz line rate). **a**) An error signal image of a scratched muscovite mica demonstrates the dominant speed induced distortions: 1. The fast turn-around of the scanner causes X-ringing which appears as waviness in height and causes positional inaccuracies. 2. Fast steps in height excite the resonances in Z-direction, causing Z-ringing, visible as ripples in flat areas that follow the shape of the step. **b**) Finite element simulation of a tube scanner shows the length extensional and lateral resonance modes associated with the major distortions. The inset figure sketches how lateral resonances can induce an apparent ΔZ in height. **c**) The scanner resonances can be separated into a lateral component in X-direction and an axial component in Z-direction acting on a mass. Due to scanner imperfections, there is cross-coupling between topography tracking and positioning.

**Figure 2:**
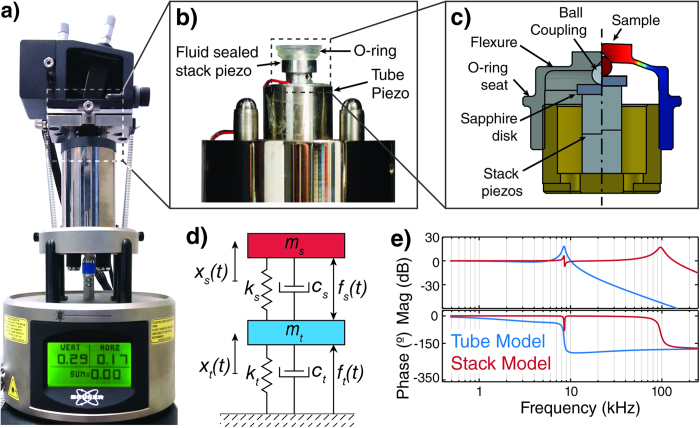
Measurement setup. **a**) Commercial atomic force microscope modified with home-built small lever head^29^,^30^ and secondary serial actuator. **b**) The secondary piezo stack actuator (two PL022.30, Physik Instrumente, Germany) inside a custom piezo housing is placed on top of the conventional piezo tube. A silicone O-ring allows imaging in fluid with a closed fluid cell. **c**) Cross-section of the stack piezo housing. A radial titanium flexure allows for sample movement while providing full fluid seal of the piezos. A sapphire disk on steel ball decouples the piezo motion from the flexure movement, while translating the piezo extension into Z-axis movement. The two pieces of the housing are joined with a fine pitch thread for piezo pre-load. **d**) Simplified schematic of the used two-actuator design, modelled as a two-body coupled oscillator. **e**) Frequency domain analysis of the model predicts simple harmonic oscillator behaviour for the large tube actuator, whereas the response of the faster stack piezo actuator shows a primary resonance and couplings into the tube actuator, causing frequency dependent distortions.

**Figure 3:**
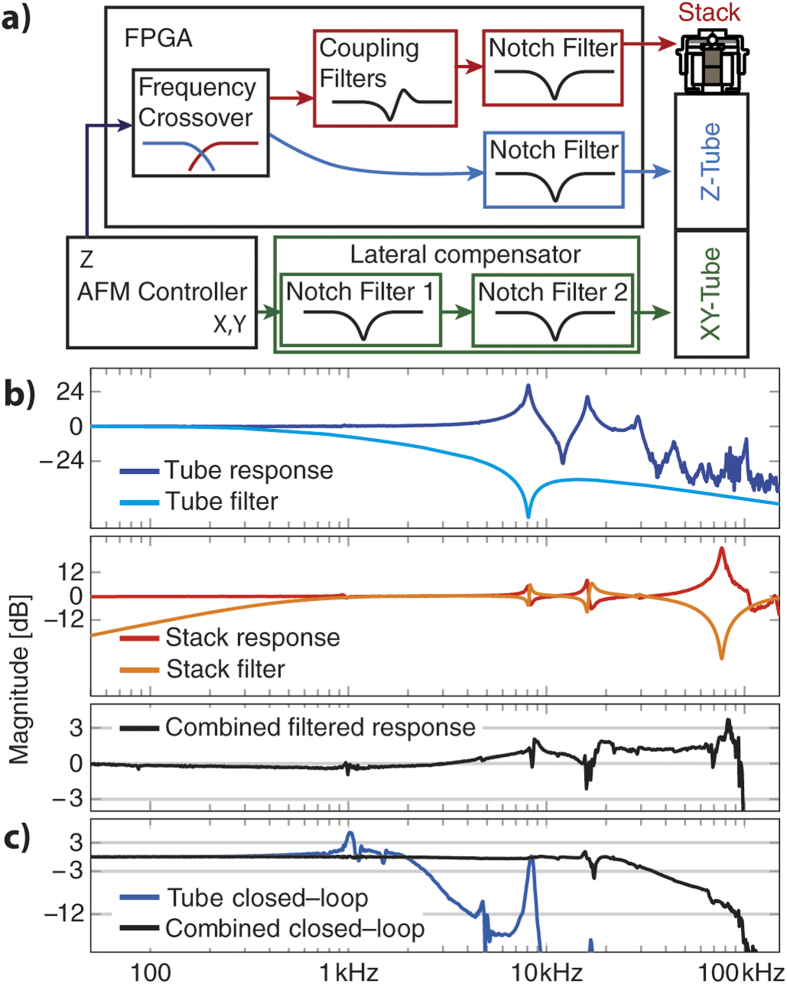
Control schematic of the two-actuator scanner with resonance suppression. **a**) A frequency crossover splits the incoming Z-signal into slow components which are sent to the long range but slow tube while high—frequency movement is executed with the short-range but fast stack actuator. The filters to suppress scanner dynamics are designed as a series of elementary filter blocks, compensating either a resonant coupling (coupling filters) or a primary resonance (notch filter). Lateral scan signals are notch-filtered for the first and optionally the second lateral resonant mode^31^. **b**) Frequency domain responses of the tube actuator (dark blue) and the stack actuator (red) with their associated filters (light blue and orange respectively) as well as the combined Z-response of the system. Due to crossover attenuation, only the first resonance of the tube needs to be notched, however coupling from the stack needs to be compensated for all modes below the stack resonance. The combined small—signal response is flat within 3 dB with hardly any peaking at the end, allowing for controller bandwidths close to the bandwidth of the scanner itself. **c**) Non-peaking small—signal closed loop transfer function of the scanner using just the tube (blue) and the combination of tube and stack actuators. The roll-off starting at 20 kHz is due to high delay of our digital PID controller.

Lateral compensation (X-Y ringing) requires only a notch filter per resonance; usually only the fast scan direction requires compensation. The method for indirect system identification described by Burns et al.^31^ is used to determine the model parameters. The resonance frequency and damping is extracted from the ring down in the X-Y to Z coupling after turnaround (compare vertical waves in Fig. 1a).

**Figure 4:**
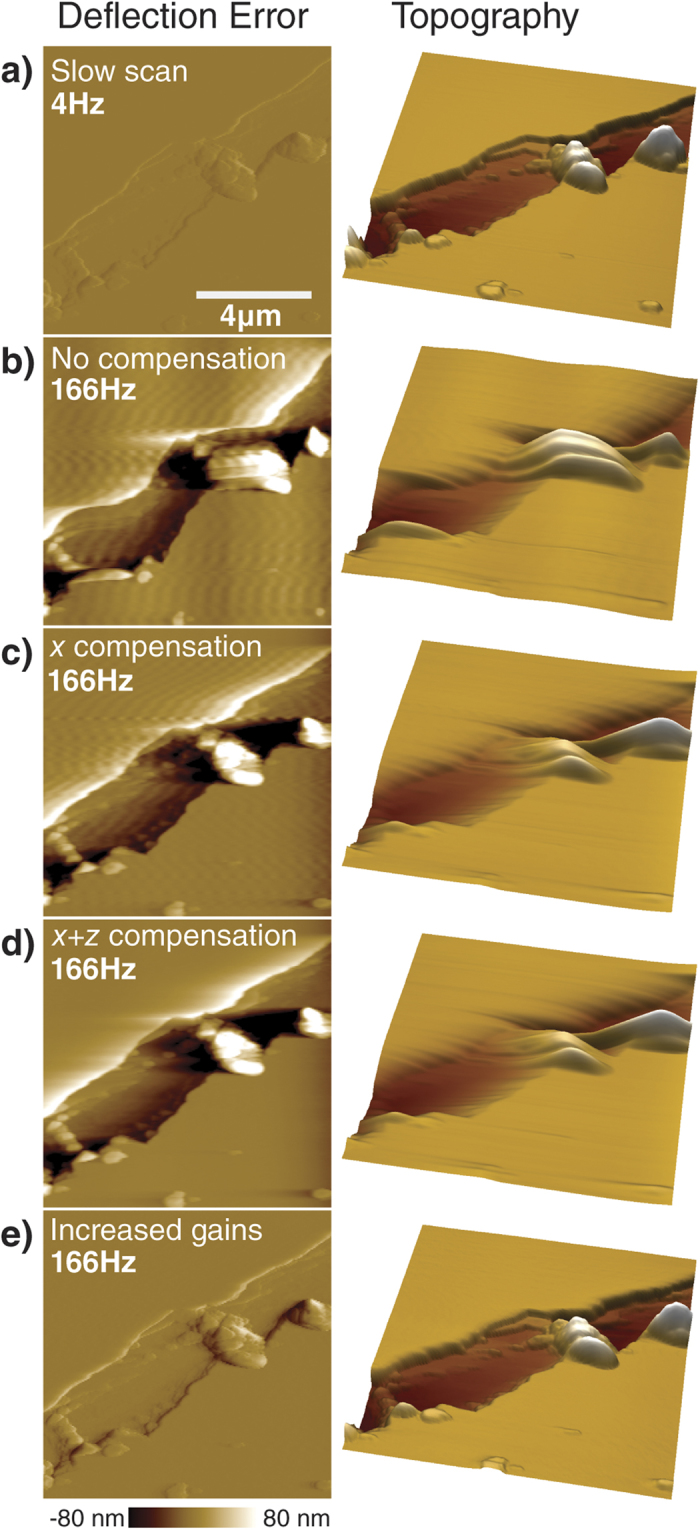
Demonstration of the effect and performance of the individual filters on imaging. **a**) The slow scan shows good tracking and no visible artefacts. **b**) Without any compensating filters the previously described distortions are clearly visible when increasing scan speed by a factor of 40. **c**) Removing lateral resonances restores the positional accuracy and removes the waviness in height. **d**) Additionally, turning on dual actuation removes the step ringing. **e**) With all filters active, the additional free scanner bandwidth can be used to increase the feedback gains, restoring the tracking close to the original slow scanning performance. All images have the same scaling.

To illustrate the effect of the individual filters, we have imaged a scratched muscovite mica sample at a moderate 4 Hz line rate in contact mode AFM with the feedback gains set to just avoid feedback oscillation. At these conditions we increase the line rate to 166 Hz (see Fig. 4a,b). While continuously scanning at high rate, we first enable the X-compensation to supress the lateral resonances, removing positional artefacts and the waviness in height (Fig. 4c). Enabling the interoperating two actuators removes the Z-ringing (Fig. 4d). Suppressing the Z-resonances and bypassing their associated phase delays allows the use of much more aggressive PID settings. Increasing the feedback bandwidth by choosing higher PID gains allows us to use the full bandwidth of the scanner and restore the imaging output to nearly the same quality as the original slow imaging (Fig. 4e).

## Results

### High—speed imaging of lipid detachment by charged dendrimers

To validate the capabilities of our system, we chose to study the interaction of positively charged dendrimer nanoparticles with lipid bilayers. The interaction of nanoparticles with biological membrane is an interesting question, both from an environmental, toxicological perspective^40^,^41^,^42^ as well as from a potentially therapeutic perspective^43^,^44^,^45^,^46^. In both cases, how the nano-particles interact with the membrane is critical in determining their ultimate effect. The combination of supported lipid bilayers and AFM provide an ideal combination to directly observe the interactions of nano-particles with the membrane in a simplified, controllable setting^47^,^48^,^49^,^50^. The mixed DLPC-DPPC bilayer used in our system allows for direct observation of the influence of lipid phase on the interaction with the positively charged nanoparticles, since both DLPC and DPPC have the same chemical head group, but have different melting temperatures. At room temperature (where we performed our experiments), the DLPC is in the fluid phase and the DPPC is in the gel phase. To avoid excessive heating of the cantilever and sample we reduced the laser power of the 635 nm laser diode in our custom AFM head^29^,^30^ to just above the lasing threshold.

**Figure 5:**
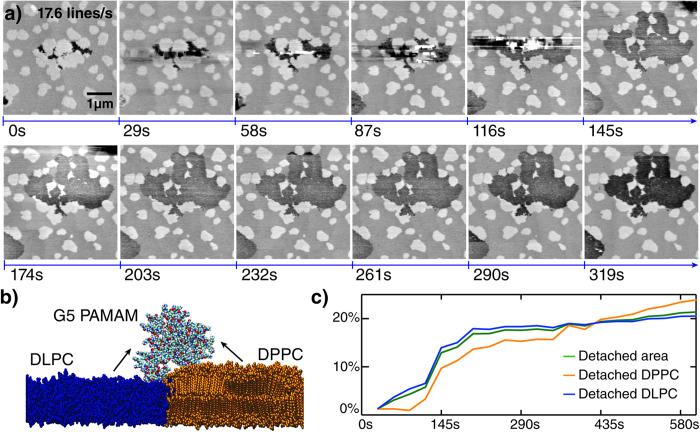
Detachment of supported lipid bilayer by polycationic dendrimers. **a**) Image sequence showing the originally intact lipid bilayer which gets disrupted after injecting G5 PAMAM dendrimer in solution. **b**) The high surface charge of the fifth generation poly-(amido-amine) (G5 PAMAM) dendrimer nanoparticles disrupts the DLPC/DPPC lipid bilayer (fluid DLPC is depicted in blue and gel DPPC is depicted in orange). **c**) Detachment curve measured from processed high—speed AFM measurements.

This experiment is also an ideal test system for HS-AFM, because any remaining, uncompensated dynamics would result in nanometre sized height distortions that would clearly exceed the height difference between DLPC and DPPC (ca. 0.12 nm). The delicate structure of the bilayer requires excellent force control, which in turn requires a high Z-bandwidth. In previous AFM experiment on this system, the long time between frames (2–5 min) has limited the understanding of the mechanism, as the whole interaction happens inside one image. HS-AFM has been sucessfully used to study dynamic effects on lipid bilayers in the past^51^,^52^ and allows us to dramatically reduce the time between images in this experiment (29 seconds, 512 × 512 pixels, 17.6 lines/s) while scanning a relatively large area (5 *μ*m). For this experiment, feedback was limited by the bandwidth of the cantilever in fluid (ca. 30 kHz), rather than the scanner itself. The additional temporal resolution has lead to the discovery of two key mechanistic results. First, the fluid DLPC is easier to disrupt than the gel DPPC. Initial defects appear only in the fluid region (DLPC), and until ca. 400 seconds after nanoparticle addition, the amount of DLPC removed outpaces that of DPPC. The second result is that the DPPC removal occurs almost exclusively after the removal of the surrounding DLPC proceeding from the destabilized edge inward. This highlights that the dendrimers are more likely to disrupt already weakened areas due to a lower activation barrier. Both of these observations would have been extremely difficult to make with conventional AFM because a single image could easily have taken more than 500 seconds completely obscuring these features. Our initial results hold promise for more mechanistic studies of membrane-nanoparticle interactions based on the measurement of kinetics and disruption patterns (Fig. 5). Permutations of charges both on the polymers and lipids will elucidate the different preferences in interaction. By adding sterols and other membrane integrating components one can probe their stabilizing and destabilizing effects. The large scan range (130 *μ*m in X and Y) also enables the imaging of rare structures in native membranes that one could otherwise never find with the small scan sizes of current HS-AFMs. To transitioning form supported bilayers to suspended bilayer structures careful force control will be even more important, to investigate the role of lipid curvature on stability.

### High—rate peak force tapping

**Figure 6:**
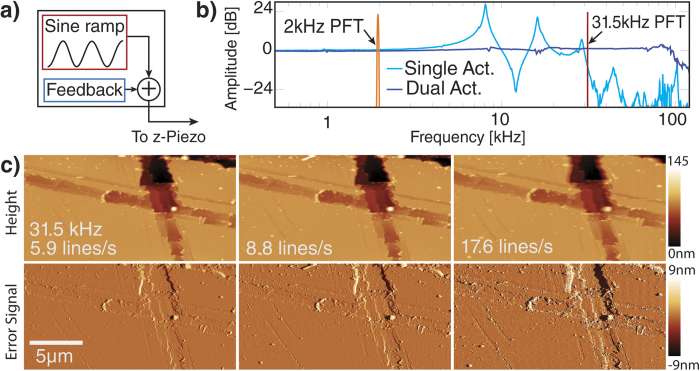
AFM imaging in high frequency peak force tapping (PFT). **a**) In peak force tapping AFM, the Z-position is additionally modulated sinusoidally and force distance curves are extracted from the cantilever deflection signal. The maximum deflection is determined for each curve and used as feedback signal for the PID, resulting in good force control. **b**) Conventional PFT operates well below the scanner resonance (2 kHz) to avoid resonant effects. Using our dual actuation system we can operate peak force tapping well beyond the first two resonances of the tube scanner at up to 31.5 kHz. **c**) Imaging with a 31.5 kHz PFT rate at different line rates. At 5.9 lines/s imaging is done with 2.7 taps/pixel, at 8.8 lines/s with 1.8 taps/pixel and at 17.6 lines/s with 0.9 taps/pixel. Even at less than a tap per pixel an image can be recorded. All images have the same scaling.

The need for mechanical property characterization at the nanoscale has accelerated the use of off-resonance AFM modes in recent years^53^,^54^,^55^,^56^ and offers very promising applications to cell biology^57^,^58^. In these modes, the cantilever is moved up and down relative to the sample in a controlled trajectory. One implementation of the off-resonance modes, peak force tapping (PFT), uses scanner motion to perform approach—retract curves at rapid rates. A sinusoidal motion is superimposed on the feedback signal (Fig. 6a). The peak interaction force is extracted from each curve and used as feedback signal for the PID controller. This approach has the benefit that it offers very good force control, as well as ease of use^53^,^59^,^60^. Since it does not depend on exciting the cantilever resonances it is well suited for automated image acquisition and gain setting. Additionally, on—line model fitting can be used to extract and display materials properties such as Young’s modulus or adhesion in real time.

With conventional scanners, the PFT rate is limited by the scanner resonance, as the frequency of the sinusoidal modulation has to stay well below the scanners resonance frequency to avoid distorted motion (see orange arrow in Fig. 6b). Most commercial systems run at 2 kHz PFT rate, high—end systems can reach up to 8 kHz rates, which makes PFT generally slower than contact mode or tapping modes. The imaging speed in PFT is directly dependent upon the PFT rate, as the number of taps per pixel defines the feedback performance (Fig. 6b).

Using the fast flat response of our two—actuator scanner, we were able to operate at a 31.5 kHz PFT rate, enabling much higher line rates (6c). Even at only one tap per pixel an image can still be aquired at reasonable quality. We are currently limited by the 500 kHz sampling rate of our controller, as the peak force extraction starts to become unreliable at roughly 16 points per curve. Due to the flat frequency response of our scanner we anticipate that the system could be operated at PFT rates up to 85 kHz once faster controllers become available.

One eventual limitation for high speed peak force tapping with this implementation is that the required current for the piezo increases drastically as the peak-force frequency increases. The absolute calculated peak currents that our system is capable of are around 2 A. However, during operation in QNM this will typically stay below 100 mA. Since the majority of the power is not dissipated into the piezo, but into the amplifier, we have not observed any problematic heating of the piezo at the currently implemented rates.

## Discussion

The performance of the system both in terms of scan range and tracking bandwidth show great promise for the dynamic imaging of biological samples. In particular the ability to track the small, high spatial frequencies in a sample that often exist on the background of large amplitude, low spatial frequency topography enables the use of HS-AFM on a much broader range of applicable samples. Imaging in fluid is shown to enable experiments on delicate biologically relevant systems. This approach requires the recording and processing of system dynamics, as sample weight and size can vary, especially when imaging in liquid. However since the complete identification is done directly on the cantilever deflection, the creation of the filters can be completely automated and in real-time, as well as made completely transparent to the end—user. The sample surface in the current design is small and comparable with other high—speed systems (some mm^2^), but could potentially be extended by using different piezo housings. The design is limited in range at high speeds, as fast changes can only be effected with the ±750 nm range of the stack piezo, while the tube scanner is primarily used to compensate large background topography, drift and sample tilt. As the higher spatial frequencies of most samples are small in magnitude we have not observed problems with tracking due to the limited range. One significant drawback of using two different actuators is that conventional hysteresis compensation techniques are not equipped to deal with two very distinct hysteresis behaviours, such as a stack piezo actuator in comparison to a tube piezo. This is not as problematic for biological imaging, but can be a problem for nanometrology applications, where better than 1% accuracy is required. Finally, the current implementation does not yet take into account resonant couplings between Z and X—Y.

Future work on the design should include cross compensation between the filters, as Z to X—Y coupling can create artefacts at very large steps. The compensation could be implemented without the need for any additional system characterization, because the X—Y dynamics are already identified for lateral compensation. Existing hysteresis models could be adapted into the control system to correct for aberrations. Alternatively, a Z sensor could be used to map the real position of the sample.

Independent of further development on the design, this system is well equipped to be used in research on larger—scale microbiological systems, such as membrane dynamics, both supported and *in vivo*.

## Methods

### AFM setup

The microscope used was a modified Bruker Multimode 8 with a home built small cantilever compatible head. The scanner that was modified was a Bruker J tube scanner. Signals were accessed with a Bruker Signal Access Module III. Necessary signal level adjustment between filters and microscope were done with homebuilt wideband scaling amplifiers. Piezo tube signals were amplified with an externalized Nanoscope 5 high—voltage amplifier, stack signals with a wideband piezo amplifier (Techproject, Austria).

### Filter design

Transfer functions were recorded with a digital lock-in amplifier (Anfatec E-204) directly by AFM in contact mode using a 10 nm excitation signal. The data was then fitted to the models and discretized in Matlab. The filter coefficients were loaded into a series of generic filters implemented as second order sections in Labview FPGA. The FPGA boards were a PXI-7851R for lateral suppression filters and a 7954R with a 5781 baseband transceiver for z filtering (all National Instruments). Stack and tube movement were digitally scaled to the same physical displacement per input signal.

### Lipid disruption imaging

Small unilamelar vesicle mixtures of 1,2-dilauroyl-sn-glycero-3-phosphocholine (DLPC) and 1,2-dipalmitoyl-sn-glycero-3-phosphocholine (DPPC) were prepared via sonication (both Avanti Polar Lipids Incorporated). Lipid powders were mixed before vesicle formation at a nominal molar ratio of 1:2, DLPC:DPPC. Vesicle solutions (1 mg/ml) were formed by transferring an appropriate mass of lipid into glass vials and dissolved with chloroform. The chloroform was evaporated off with dry Nitrogen gas, leaving a thin film on the glass vial. The film was hydrated with Milli-Q water (Milipore, Billerica, MA, USA), generating large multi-laminar vesicles, (LMVs). The LMVs were then sonicated with a probe sonicator (BioLogics Inc) to generate small unilaminar vesicles (SUVs). The SUVs were centrifuged to remove metal particles left from the probe sonicator. 35 *μ*l of the lipid preparation was warmed to 37 °C and deposited onto freshly cleaved mica surfaces, forming bilayers via vesicle fusion. Surfaces were allowed to incubate for at least a half hour in a humid environment at room temperature.

Imaging was done in liquid with a Bruker FastScan-C cantilever in amplitude modulation. The imaging data has been flattened and corrected for drift in post processing.

## Additional Information

**How to cite this article**: Nievergelt, A. P. *et al.* Studying biological membranes with extended range high-speed atomic force microscopy. *Sci. Rep.* **5**, 11987; doi: 10.1038/srep11987 (2015).

## Supplementary Material

Supplementary Information
