# Corrigendum: Studying biological membranes with extended range high-speed atomic force microscopy

**DOI:** 10.1038/srep21654

**Published:** 2016-03-10

**Authors:** Adrian P. Nievergelt, Blake W. Erickson, Nahid Hosseini, Jonathan D. Adams, Georg E. Fantner

Scientific Reports 5: Article number: 11987; 10.1038/srep11987 published online 07142015; updated: 03102016

The authors neglected to cite previous studies related to the control concepts of high speed atomic force microscopy in the Introduction section of this Article. These additional references are listed below as references 1 and 2, and should appear in the text as below.

“We propose a solution for a large range, high—speed system through simple modifications of a commercial system to move the sample with two different actuators in mechanical series and extending the control loop with model-based control for this scanner.”

should read:

“In line with the controls concepts introduced by Bozchalooi *et al.*^1^,^2^, we present a solution for a large range, high–speed system through simple modifications of a commercial system to move the sample with two different actuators in mechanical series and extending the control loop with model based control for this scanner.”
