# Supplementary material for: Studying biological membranes with extended range high-speed atomic force microscopy
Source: Sci Rep. 2015 Jul 14;5:11987. doi: 10.1038/srep11987 (PMC4500952; doi:10.1038/srep11987)
Supplement: Supplementary Information [file srep11987-s1.pdf]

Studying biological membranes with extended range high-speed atomic force microscopy Supplementary Information

Adrian P. Nievergelt[1,+], Blake W. Erickson[1,+], Nahid Hosseini[1], Jonathan D. Adams[1], and Georg E. Fantner[1,*]

[1]**Laboratory for Bio- and Nano-Instrumentation** , École Polytechnique Fédérale de Lausanne , Batiment BM 3109 Station 17,, 1015 Lausanne, Switzerland,
[*]georg.fantner@epfl.ch
[+]these authors contributed equally to this work

Introduction

The individual steps of measuring and implementing filters for actuation with two piezos as shown in this paper are explained here.

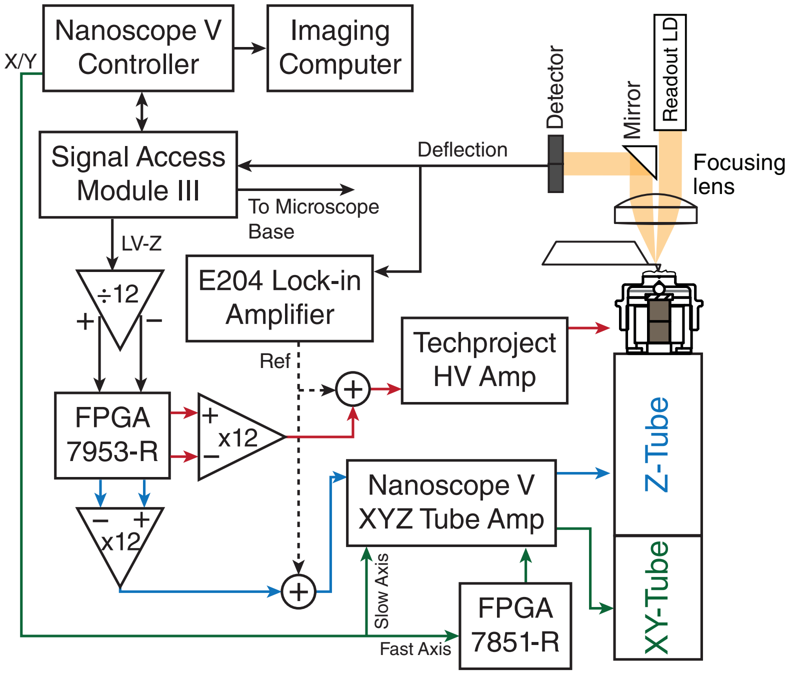

Figure 1. Detailed wiring diagram for the two–actuator AFM that is presented in this paper. The dashed lines from the lock–in amplifier denote temporary connections that are required for system identification.

System identification

Since all filters used in our implementation are model based, the transfer functions of the x/y and both z actuators have to be recorded. As the system response is extracted directly from the cantilever deflection, the AFM is needs to be engaged on the

surface in contact mode for all system identification measurements. The feedback gains should be set as low as possible, so the surface is still tracked, but all signals of interest are represented in the cantilever deflection.

The two Z actuators (stack and tube) are each individually swept with a network analyser or a lock–in amplifier with the ability to record transfer functions. The reference signal is added onto the low voltage signal of the respective actuator before amplification and the resulting motion recorded as a transfer function. The transfer functions are normalized by the physical movement, such that they contain the sensitivity of each actuator.

As described by Burns et al.[1] the X-Y dynamics can be recorded by using the X-Y to Z coupling (turnaround ripple) to identify the frequency and damping constants of the lateral motions using cross correlation.

Modelling of Z-couplings

The models for simple harmonic oscillator and tube coupling blocks can be used for fitting most coupled resonator systems. Using the model shown in figure 2c) in the main text, the frequency domain models can be derived with standard MIMO theory.

We first derive the equations of motion for the positions x_t of the tube and x_s for the stack actuators as

$$\begin{bmatrix} m_s & 0 \\ 0 & m_t \end{bmatrix} \begin{bmatrix} \ddot{x}_s \\ \ddot{x}_t \end{bmatrix} = M\ddot{z} = \begin{bmatrix} -k_s & k_s \\ k_s & -k_t - k_s \end{bmatrix} z + \begin{bmatrix} -c_s & c_s \\ c_s & -c_t - c_s \end{bmatrix} \dot{z} + \begin{bmatrix} f_s \\ f_t - f_s \end{bmatrix} \tag{1}$$

where m_t and m_s are the masses, c_t and c_s are the damping coefficients and k_t and k_s are the spring constants of the tube and stack respectively. By rewriting (1) we find the state space representation

$$dotx = Ax + Bu, \quad y = Cx + D \tag{2}$$

of the MISO system with the matrices

$$x = \begin{bmatrix} x_s \\ x_{s,1} \\ x_t \\ x_{t,1} \end{bmatrix} \qquad u = \begin{bmatrix} f_s \\ f_t \end{bmatrix} \qquad A = \begin{bmatrix} 0 & 1 & 0 & 0 \\ \frac{-k_s}{m_s} & \frac{-c_s}{m_s} & \frac{-k_s}{m_s} & \frac{c_s}{m_s} \\ 0 & 0 & 0 & 1 \\ \frac{k_s}{m_t} & \frac{c_s}{m_t} & \frac{-k_s-k_t}{m_t} & \frac{-c_s-c_t}{m_t} \end{bmatrix} \tag{3}$$

$$B = \begin{bmatrix} 0 & 0 \\ \frac{1}{m_s} & 0 \\ 0 & 0 \\ \frac{-1}{m_t} & \frac{1}{m_t} \end{bmatrix} \qquad C = \begin{bmatrix} 1 & 0 & 0 & 0 \\ 0 & 0 & 0 & 0 \end{bmatrix} \qquad D = 0 \tag{4}$$

which takes the piezo extension forces which are roughly proportional to the applied voltages as inputs and outputs the scalar position $y = x_s$ at the top of the stack. Using MIMO system analysis we find the transfer function matrix as per

$$H(s) = \begin{bmatrix} H_s(s) & H_t(s) \end{bmatrix} = \frac{Y(s)}{U(s)} = \frac{C\,\mathrm{adj}(sI - A)B}{\det(sI - A)}. \tag{5}$$

The response of the sample position to a change in the tube acts as per its transfer function

$$H_t(s) = \frac{c_s s + k_s}{m_t m_s s^4 + (m_t c_s + m_s(c_t + c_s))s^3 + (m_t k_s + m_s(k_t + k_s) + c_t c_s)s^2 + (c_t k_s + c_s k_t)s + k_t k_s} \approx \frac{1}{(m_t + m_s)s^2 + c_t s + k_t} \tag{6}$$

like a SHO with added mass if we assume that $k_s \gg k_t$. When actuating the stack actuator however we get

$$H_s(s) = \frac{m_t s^2 + c_t s + k_t}{m_t m_s s^4 + (m_t c_s + m_s(c_t + c_s))s^3 + (m_t k_s + m_s(k_t + k_s) + c_t c_s)s^2 + (c_t k_s + c_s k_t)s + k_t k_s} \tag{7}$$

which can be understood as coupling into the softer tube with a higher frequency resonance of the stack actuator. Modeling a complex resonant mechanical system such as this as coupled spring dampener system does not take into account higher eigenmodes. However the individual SHO models as well as the coupling models can be individually fitted to the data and combined in series to compensate the complete dynamics of the more complicated system.

Fitting the models to the data and transfer to hardware

To implement model inversion filters the filter blocks (notch filters and coupling filters) have to be fitted to the respective dynamics on the transfer functions. We use a Matlab script using System Identification, Curve Fitting and Signal Processing toolboxes to generate the filters. The models are fitted to the data in continuous form, then discretized into ZPK form with standard functions. Each coupling and each SHO resonance is fitted separately and the individual blocks are afterwards combined in series to a total transfer function. The filters are implemented as a series of second order sections.

Finally, the generated filters have to be loaded onto the FPGA hardware. This is done in our case with a front panel written in LabView. The complete signal routing as has been used in this implementation can be seen in fig. 1. As the Nanoscope V controller does not have direct signal access, a Signal Access Module has to be used. The X-scan signal is output on the frontpanel of the controller. The 7953-R FPGA we use for it's high speed (100 MS/s with 40 MHz analog bandwidth NI 5781 baseband transceiver) is limited to 4 V peak to peak differential input. We use a homebuilt 600 MHz single-ended to differential scaling amplifier as interface. The 7953-R FPGA runs at 2 MHz sample rate, while the loop rate is set to 100 MHz. A set of upscaling amplifiers restores the Nanoscope 24 V peak to peak single ended voltage levels, which are then sent to their respective amplifiers. The slower 7851-R FPGA can handle the higher voltage levels and does not need any extra interfacing hardware. It was set to 400 kHz sample rate with a loop rate is of 80 MHz. The tube piezos are driven with an isolated high–voltage board from a Nanoscope V controller. The stack needs a special high voltage amplifier (Techproject, Austria) due to the higher capacitive load.

To peform the system identification a sweepable lock–in amplifier (Anfatec E204, Germany) was used. The generated reference signals are added onto the FPGA output with homebuilt analog adders while the FPGA is set to passthrough mode (no filters).

References

1. Burns, D. J., Youcef-Toumi, K. & Fantner, G. E. Indirect identification and compensation of lateral scanner resonances in atomic force microscopes. *Nanotechnology* **22**, 315701 (2011). URL http://iopscience.iop.org/0957-4484/22/31/315701.
